# Sex and survival in non-small cell lung cancer: A nationwide cohort study

**DOI:** 10.1371/journal.pone.0219206

**Published:** 2019-06-27

**Authors:** Cecilia Radkiewicz, Paul William Dickman, Anna Louise Viktoria Johansson, Gunnar Wagenius, Gustaf Edgren, Mats Lambe

**Affiliations:** 1 Department of Medical Epidemiology and Biostatistics, Karolinska Institutet, Stockholm, Sweden; 2 Department of Oncology, Södersjukhuset, Stockholm, Sweden; 3 Cancer Registry of Norway, Oslo, Norway; 4 Cancer Theme, Karolinska University Hospital, Stockholm, Sweden; 5 Department of Medicine Solna, Clinical Epidemiology Division T2, Karolinska Institutet, Stockholm, Sweden; 6 Department of Cardiology, Södersjukhuset, Stockholm, Sweden; 7 Regional Cancer Center Uppsala-Örebro, Uppsala, Sweden; Catalan Institute of Oncology - Bellvitage Biomedical Research Institute (ICO-IDIBELL), SPAIN

## Abstract

**Aim:**

To in detail delineate sex differences in non-small cell lung cancer outcome and investigate possible underlying drivers.

**Methods:**

We performed a nationwide, population-based cohort study using data on all incident cases of lung squamous cell carcinoma (n = 10,325) and adenocarcinoma (n = 23,465) recorded in the Swedish Lung Cancer Register in 2002–2016. Flexible parametric models were applied to compute adjusted female-to-male hazard ratios (aHRs) and standardized survival proportions over follow-up including age, calendar year, education, marital status, birth country, health care region, performance status, smoking history, comorbidities, and tumor location in the final model.

**Results:**

Women presented with better performance status, were younger, and more often never-smokers. Women with adenocarcinoma also had lower comorbidity burden, less advanced stage, and were more often *EGFR* positive. Men with adenocarcinoma had a consistently poorer lung cancer-specific survival across stage; HR 0.69; 95% CI 0.63–0.76 (stage IA-IIB) to 0.94; 95% CI 0.88–0.99 (stage IIIB-IV), remaining largely unchanged after adjustments; aHR 0.74; 95% CI 0.66–0.82 to 0.84; 95% CI 0.81–0.87. The same pattern was observed in squamous cell carcinoma, except in stage IIIA disease, where we found no sex differences in survival.

**Conclusions:**

Men with non-small cell lung cancer have a consistently poorer prognosis, even after careful adjustments for a wide range of prognostic factors. While the pattern was similar in both squamous cell and adenocarcinoma, it was larger and more consistent in the latter.

## Introduction

Converging evidence from studies conducted in different geographic regions demonstrate that men with non-small cell lung cancer (NSCLC) have a poorer prognosis compared to women [1–11]. The inferior male survival in NSCLC appears to be consistent over calendar time and across the two major histological types; squamous cell and adenocarcinoma. It has become increasingly apparent that these two subtypes represent separate clinical entities with different epidemiology, treatment, and prognosis [12].

Prognostic factors in NSCLC encompass stage, tumor location, number of examined lymph nodes, nodal station involvement, tumor biology and molecular characteristics (histology, grade, proliferation rate, pleural and vascular invasion, mutational status), number and location of metastasis, treatment, age, smoking history, socioeconomic status, ethnicity, comorbidity, performance status, presence of pulmonary symptoms, and weight loss [1, 2, 5, 8, 13–16]. The underlying reasons behind the male excess mortality remain incompletely understood [6, 17, 18].

The aim of the present study was firstly to in detail characterize and quantify sex differences in non-small cell lung cancer outcome. Secondly, to investigate a wide range of possible factors contributing to these differences.

## Materials and methods

### Data sources and covariates

We performed a population-based cohort study using prospectively collected data from the Lung Cancer DataBase Sweden (LcBaSE) from 2002 until 2016. LcBaSE is a research database containing detailed patient-level data from several nationwide registers, including the Swedish National Lung Cancer Register (NLCR), the Swedish Cancer Register, the National Patient Register, the Prescribed Drug Register, the Longitudinal Integration Database for Health Insurance and Labor Market Studies (LISA), the Cause of Death Register, and the Total Population Register [19, 20]. The national registration number, a personal identifier assigned to all residents of Sweden, enabled individual-level record linkages between registers [21].

The NLCR started in 2002 and aims to include all Swedish residents diagnosed with invasive lung cancer according to the International Classification of Diseases for Oncology code C34. Postmortem diagnoses, carcinoma in situ, tracheal, and pleural tumors are not included. The completeness compared to the mandatory Swedish Cancer Register is 96% [19, 20]. The NLCR records clinicopathological factors at diagnosis as well as diagnostic investigations and primary treatment [19]. We applied the Elixhauser method to assess comorbidity burden using data on main and secondary diagnoses at discharge from hospital from the National Patient Register and data on other malignancies from the Swedish Cancer Register, 15 years—1 month before lung cancer diagnosis [20, 22]. Coding was according to the International Classification of Diseases revision 9 (year 1987–1996) and 10 (year 1997–2016). The Elixhauser approach is considered valid and reliable and was applied in its original form, treating the 31 comorbidity groups as independent, binary variables [22, 23]. Additional measures of comorbidity, including number of medications prescribed 6–18 months before NSCLC diagnosis, grouped according to the first three positions of the Anatomical Therapeutic Chemical Classification, number of in- and outpatient visits 6–18 months before diagnosis, and the Charlson Comorbidity Index, were explored. Educational level and marital status were acquired from the LISA database. Date and cause of death were retrieved from the Cause of Death register. Information on emigration was ascertained from the Total Population Register.

Inclusion criteria included a first record of lung cancer, squamous cell or adenocarcinoma histopathology, year of diagnosis 2002–2016, and age at diagnosis 20 years or older (n = 34,003). More rare histologic types of non-small cell lung cancer (large cell carcinomas, carcinoid tumors, adenosquamous carcinomas, sarcomatoid carcinomas, salivary gland-type tumors, and unclassified carcinomas) were not included. Histologically unverified cases (n = 110) and cases with missing date of birth (n = 102) were excluded. This way, the final study population encompassed 33,790 men and women with NSCLC (S1 Fig). Staging was based on the tumor-node-metastasis (TNM) system by the American Joint Committee on Cancer, the 6th revision up until 2010 when the 7th revision was introduced in clinical practice [24].

### Statistical analyses

All analyses were stratified on histology. Analyses on treatment and mortality were additionally stratified into early (IA-IIB), locally advanced (IIIA), and late (IIIB-IV) stage disease, based on treatment guidelines.[25] Numbers and percentages of missing data were low (0–3.7%) and similar between sexes; the missing indicator approach was applied to not exclude these cases from multivariable analyses.

The frequency of clinicopathological characteristics at diagnosis, including calendar period, level of education, age, ECOG performance status, smoking history, Elixhauser comorbidities, TNM stage, and *EGFR* mutation status (advanced stage adenocarcinoma, years 2010–2016) were calculated for men and women. The Pearson chi-square test was applied to compare distributions.

To quantify sex differences in diagnostic and treatment intensity, we used logistic regression with robust standard errors to compute odds ratios (OR) with 95% confidence intervals (CI) for each modality. Treatment-on-time was defined as treatment initiation or decision within 28 days from referral to specialist care. For each diagnostic/treatment modality we ran two models, the first included age and year of diagnosis (OR), and the second (aOR) also incorporated educational level, marital status, birth country, health care region, ECOG performance status, smoking history, Elixhauser comorbidities, TNM stage, and primary tumor location.

Date of diagnosis was defined as the date of histopathological or cytological sample collection. Survival time was calculated from date of diagnosis until date of death, emigration, or end of follow-up (December 31, 2017), whichever occurred first. Lung cancer-specific death was defined as C34 according to the 10th revision of the International Classification of Diseases. Flexible parametric models were applied to estimate female-to-male hazard ratios (HR) with 95% confidence intervals (CI) [26]. As a sensitivity analysis we explored the effects of several confounders by including them sequentially, evaluating the effect on the HR. All survival models included age and year of diagnosis (HR), the fully adjusted model (aHR) also included level of education, marital status, birth country, health care region, ECOG performance status, smoking history, Elixhauser comorbidity categories, TNM stage, and primary tumor location. We performed a subgroup analysis of patients diagnosed with lung adenocarcinoma in 2010–2016 tested for *EGFR*, and fitted a third model, additionally adjusted for *EGFR* mutational status. Further sensitivity analyses were employed to explore effect modification and model fit including interaction between sex and selected covariates.

In addition to Kaplan-Meier survival curves, we estimated standardized survival proportions for men and women over follow-up including the absolute difference (women-men) in survival at 1, 3 and 5 years after diagnosis, with 95% CI, using fully-adjusted flexible parametric models [26]. This approach predicts one survival curve for each individual in the strata under study and averages these to create two standardized survival curves were the only differences is that in one everyone is exposed (male sex) and in the other one unexposed (female sex) [27]. The baseline hazard function was fitted using restricted cubic splines with 5 degrees of freedom, generating 4 interior knots placed at the 20th, 40th, 60th, and 80th percentile. To allow for non-proportional hazards, the time-dependent effect of sex was fitted using restricted cubic splines with 3 degrees of freedom.

All statistical analyses were performed using Stata Intercooled version 15.1 (StataCorp LP). The Stata commands stpm2 and stpm2_standsurv were used when applying flexible parametric models [26]. This study was approved by the regional Ethical Review Committee in Stockholm (2012/1162-31/4; 2016/1137-32; 2017/445-32).

## Results

We identified 33,790 men and women with a diagnosis of lung squamous cell (n = 10,325) or adenocarcinoma (n = 23,465). Table 1 summarizes clinicopathological factors by histology and sex (S1 Table summarizes all investigated factors). While the incidence of lung adenocarcinoma increased overall, the incidence of squamous cell carcinoma decreased in men and increased in women. In both subtypes, women had a higher education, were more often never-smokers, younger, and presented with better performance status at diagnosis, compared to men. In adenocarcinoma, women also had a lower comorbidity burden, presented with less advanced stage, and more often had *EGFR* positive tumors. In general sex differences were less pronounced in squamous cell than in adenocarcinoma.

**Table 1:** Clinical characteristics at diagnosis.

	Squamous cell carcinoma					Adenocarcinoma				
	Men		Women		p-value	Men		Women		p-value
	n	%	n	%		n	%	n	%	
All cases	6556	100.0	3769	100.0		10795	100.0	12670	100.0	
**Calendar period**										
2002–2006	2225	33.9	1119	29.7		2695	25.0	2882	22.7	
2007–2011	2200	33.6	1262	33.5		3671	34.0	4241	33.5	
2012–2016	2131	32.5	1388	36.8	<0.001	4429	41.0	5547	43.8	<0.001
**Education**										
Low	3235	49.3	1751	46.5		4559	42.2	4808	37.9	
Middle	2383	36.3	1495	39.7		4230	39.2	5427	42.8	
High	762	11.6	452	12.0		1809	16.8	2273	17.9	
missing	176	2.7	71	1.9	<0.001	197	1.8	162	1.3	<0.001
**Age**										
0–59	659	10.1	445	11.8		1551	14.4	2344	18.5	
60–69	2002	30.5	1168	31.0		3702	34.3	4571	36.1	
70–79	2711	41.4	1521	40.4		3966	36.7	4106	32.4	
80–89	1148	17.5	612	16.2		1501	13.9	1562	12.3	
90+	36	0.5	23	0.6	0.04	75	0.7	87	0.7	<0.001
**ECOG performance status**										
0	1126	17.2	629	16.7		2501	23.2	3514	27.7	
1	2528	38.6	1553	41.2		3998	37.0	4829	38.1	
2	1502	22.9	875	23.2		2183	20.2	2276	18.0	
3	886	13.5	465	12.3		1256	11.6	1264	10.0	
4	272	4.1	139	3.7		455	4.2	448	3.5	
missing	242	3.7	108	2.9	0.02	402	3.7	339	2.7	<0.001
**Smoking history**										
Smoker	3120	47.6	1976	52.4		4109	38.1	5120	40.4	
Former smoker	3160	48.2	1470	39.0		5336	49.4	4957	39.1	
Never-smoker	165	2.5	253	6.7		1147	10.6	2384	18.8	
missing	111	1.7	70	1.9	<0.001	203	1.9	209	1.6	<0.001
**Elixhauser comorbidities**										
0	2261	34.5	1386	36.8		4196	38.9	5557	43.9	
1–2	2628	40.1	1469	39.0		4171	38.6	4842	38.2	
3–4	1151	17.6	628	16.7		1703	15.8	1617	12.8	
5+	516	7.9	286	7.6	0.13	725	6.7	654	5.2	<0.001
**Stage**										
IA-IIB	1827	27.9	1125	29.8		2470	22.9	3387	26.7	
IIIA	892	13.6	519	13.8		762	7.1	987	7.8	
IIIB-IV	3703	56.5	2069	54.9		7398	68.5	8143	64.3	
missing	134	2.0	56	1.5	0.04	165	1.5	153	1.2	<0.001
*EGFR* mutation*										
Positive	-	-	-	-		221	5.5	472	10.3	
Negative	-	-	-	-		1880	46.5	2159	47.1	
Inconclusive	.	-	-	-		157	3.9	210	4.6	
Pending	-	-	-	-		23	0.6	34	0.7	
not tested	-	-	-	-		1765	43.6	1713	37.3	<0.001

*Stage IIIB-IV, year of diagnosis 2010–2016

When comparing diagnostic intensity as well as if primary treatment was decided and/or commenced within three weeks from referral to a specialist unit, in men and women (S2 Table), absolute differences were minor and statistically significant only for a few procedures.

Sex differences in treatment are presented in Table 2. Investigated treatment modalities included surgery and hypofractionated radiotherapy (2007–2016) in early stage; surgery, chemo-radiotherapy (2007–2016), and radiotherapy in locally advanced stage; and systemic therapy, radiotherapy, and chemo-radiotherapy (2007–2016) in late stage disease. We found no evidence of unequal treatment of Swedish men and women with NSCLC, with the exception of chemo-radiotherapy being marginally more common in men with late stage adenocarcinoma (20.2 vs 17.9%, aOR 1.14, 95% CI 1.01–1.29).

**Table 2:** Treatment intensity.

**Squamous cell carcinoma**							
	**Men**			**Women**			
	n	%	OR (95% CI)	n	%	OR (95% CI)^1^	aOR (95% CI)^2^
**Stage IA-IIB**							
Surgery	1063	58.2	1.00 (ref.)	683	60.7	1.04 [0.88,1.22]	0.92 [0.74,1.15]
Hypofractioned radiotherapy*	137	11.2	1.00 (ref.)	108	13.4	1.25 [0.95,1.65]	1.16 [0.82,1.63]
**Stage IIIA**							
Surgery	94	10.5	1.00 (ref.)	58	11.2	1.05 [0.74,1.50]	1.05 [0.70,1.58]
Chemo-radiotherapy*	287	45.5	1.00 (ref.)	177	44.1	0.94 [0.72,1.22]	1.02 [0.73,1.43]
Radiotherapy	242	27.1	1.00 (ref.)	117	22.5	0.88 [0.68,1.15]	0.89 [0.65,1.23]
**Stage IIIB-IV**							
Chemotherapy	2149	58.0	1.00 (ref.)	1240	59.9	1.06 [0.94,1.19]	1.08 [0.92,1.25]
Radiotherapy primary tumor	572	15.4	1.00 (ref.)	328	15.9	1.11 [0.95,1.30]	1.11 [0.94,1.31]
Chemo-radiotherapy*	488	20.2	1.00 (ref.)	254	17.9	0.85 [0.73,1.01]	0.89 [0.73,1.08]
**Adenocarcinoma**							
	**Men**			**Women**			
	n	%	OR (95% CI)	n	%	OR (95% CI)^1^	aOR (95% CI)^2^
**Stage IA-IIB**							
Surgery	1816	73.5	1.00 (ref.)	2626	77.5	1.11 [0.97,1.27]	0.96 [0.81,1.14]
Hypofractioned radiotherapy*	242	12.7	1.00 (ref.)	299	11.1	0.97 [0.80,1.17]	0.96 [0.76,1.20]
**Stage IIIA**							
Surgery	151	19.8	1.00 (ref.)	226	22.9	1.15 [0.91,1.46]	1.19 [0.90,1.58]
Chemo-radiotherapy*	311	50.3	1.00 (ref.)	385	47.4	0.84 [0.68,1.05]	0.86 [0.67,1.10]
Radiotherapy	130	17.1	1.00 (ref.)	137	13.9	0.78 [0.58,1.03]	0.80 [0.57,1.11]
**Stage IIIB-IV**							
Chemotherapy	4820	65.2	1.00 (ref.)	5475	67.2	1.05 [0.98,1.13]	0.97 [0.88,1.06]
Radiotherapy primary tumor	509	6.9	1.00 (ref.)	493	6.1	0.90 [0.79,1.02]	0.89 [0.77,1.02]
Chemo-radiotherapy*	551	10.1	1.00 (ref.)	716	11.6	1.14 [1.02,1.27]	1.14 [1.01,1.29]

Numbers (n), percentages (%) of male and female NSCLC patients, and female-to-male odds ratios (ORs) with 95% confidence intervals (CI), undergoing treatment by histological type and stage.

^1^Adjusted for age and calendar year of diagnosis.

^2^Additionally adjusted for level of education, marital status, birth country, health care region, ECOG performance status, smoking history, Elixhauser comorbidity categories, TNM stage, and primary tumor location.

*Year 2007–2016.

Table 3 shows numbers and percentages of lung cancer deaths in men and women as well as lung cancer-specific female-to-male HR by histology and stage. Overall, there were very small differences between the basic HR and the fully adjusted aHR. Women had a consistently better prognosis with the exception of squamous cell carcinoma stage IIIA. Sex differences in lung cancer mortality were most pronounced in early and locally advanced adenocarcinoma, aHR 0.74 (95% CI 0.66–0.82) and 0.77 (95% CI 0.67–0.88), respectively. In the subgroup analysis of lung adenocarcinomas tested for *EGFR*, the female-to-male hazard ratio remained unaltered in the models adjusted for sex differences in *EGFR* mutational status (S3 Table).

**Table 3:** Lung cancer-specific mortality.

**Squamous cell carcinoma**				
** **	n	%	HR^1^ (95% CI)	aHR^2^ (95% CI)
**Stage IA-IIB**				
Men	844	46.2	1.00	1.00
Women	448	39.8	0.81 [0.72,0.92]	0.80 [0.70,0.92]
**Stage IIIA**				
Men	599	67.2	1.00	1.00
Women	364	70.1	1.06 [0.93,1.21]	1.04 [0.90,1.21]
**Stage IIIB-IV**				
Men	3120	84.3	1.00	1.00
Women	1713	82.8	0.94 [0.88,0.99]	0.87 [0.81,0.92]
**Adenocarcinoma**				
	n	%	HR^1^ (95% CI)	aHR^2^ (95% CI)
**Stage IA-IIB**				
Men	875	35.4	1.00	1.00
Women	943	27.8	0.69 [0.63,0.76]	0.74 [0.66,0.82]
**Stage IIIA**				
Men	475	62.3	1.00	1.00
Women	602	61.1	0.81 [0.71,0.91]	0.77 [0.67,0.88]
**Stage IIIB-IV**				
Men	6167	83.4	1.00	1.00
Women	6672	81.9	0.94 [0.88,0.99]	0.84 [0.81,0.87]

Numbers (n), percentages (%) of lung cancer deaths in male and female NSCLC patients, and female-to-male hazard ratios (HRs) with 95% confidence intervals (CI) by histological type and stage.

^1^Adjusted for age and calendar year of diagnosis.

^2^Additionally adjusted for level of education, marital status, birth country, health care region, ECOG performance status, smoking history, Elixhauser comorbidity categories, TNM stage, and primary tumor location.

**Fig 1:**
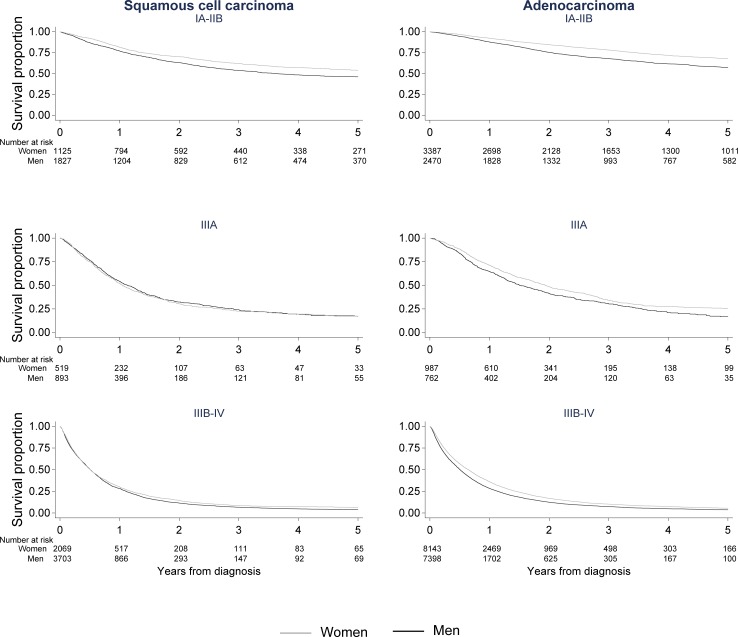
Lung cancer-specific survival (Kaplan-Meier). Lung cancer-specific survival proportion for men and women diagnosed with lung squamous cell and adenocarcinoma by follow-up time (years), stratified on stage group; IA-IIB, IIIA, IIIB-IV.

**Fig 2:**
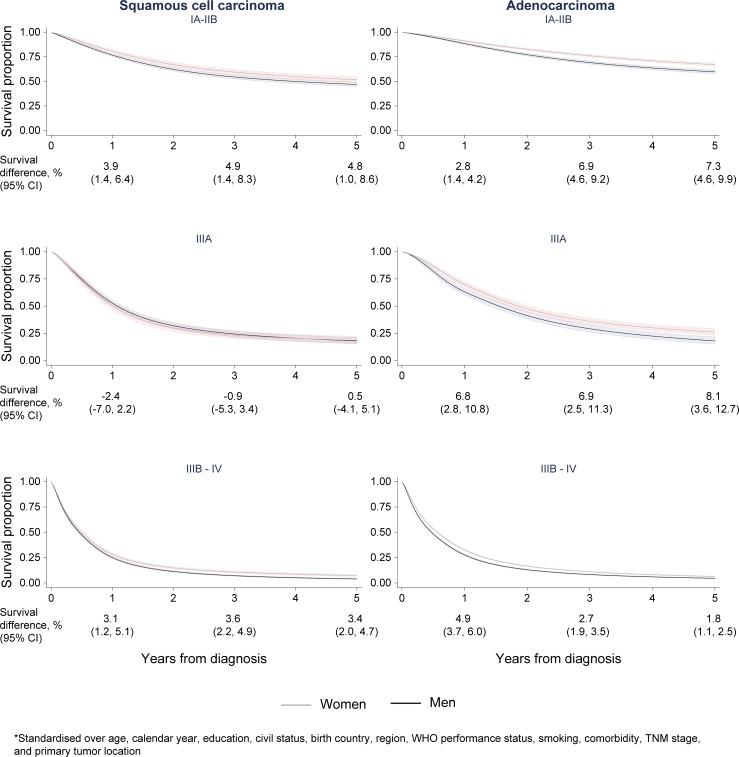
Standardized lung cancer-specific survival. Lung cancer-specific survival proportion including 95% confidence intervals for men and women diagnosed with lung squamous cell and adenocarcinoma by follow-up time (years), stratified on stage group; IA-IIB, IIIA, IIIB-IV, and standardized over age, calendar year, level of education, marital status, birth country, health care region, ECOG performance status, smoking history, Elixhauser comorbidity categories, TNM stage, and primary tumor location.

The results in Table 3 are reflected in the Kaplan-Meier curves (Fig 1). Across stage, men with pulmonary adenocarcinoma experienced poorer lung cancer-specific survival. The pattern was similar for squamous cell carcinoma, with the exception of stage IIIA disease where no sex difference in survival was observed. Similarly, the survival curves generated from multivariable, flexible parametric models (Fig 2) showed a significantly, poorer prognosis in men compared to women consistent over histology, stage, and follow-up, with the exception of squamous cell carcinoma stage IIIA. Absolute differences in survival proportions (women-men) were most pronounced 5 years after diagnosis and in early (IA-IIB) 7.3% (95% CI 4.6–9.9) and advanced (IIIA) stage 8.1% (95% CI 3.6–12.7) adenocarcinoma.

Results from the sensitivity analysis where each prognostic factor was added sequentially (S2 Fig) revealed that HR estimates remained largely unchanged following statistical adjustments for various clinicopathological factors including the Charlson Comorbidity Index. Additional sensitivity analysis exploring interaction between sex and selected covariates (S4 Table) showed that the female survival advantage in lung adenocarcinoma was remarkably stable with the exception of a possible interaction between sex and smoking history. In squamous cell carcinoma no consistent pattern in combination with superior model fit was seen when allowing for interaction in the model. Results from flexible parametric and Cox regression models were nearly identical (S5 Table).

## Discussion

Using data from a large, nationwide population-based lung cancer cohort, we demonstrate that male sex represents an independent, negative prognostic factor in NSCLC. Men with lung adenocarcinoma had a poorer prognosis than women regardless of stage. In lung squamous cell carcinoma the male survival disadvantage was demonstrated in all stage groups, except IIIA. Overall, the effect of careful adjustments for a wide range of prognostic factors was very small if any, supporting the notion that the poorer survival of men with NSCLC reflects yet to be identified sex differences in tumor biology. We found no evidence of unequal clinical management between men and women with NSCLC.

To our knowledge, this is the largest study to date addressing sex differences in NSCLC management and outcome covering individual-level data on a comparable quantity and range of ascertained covariates. The Swedish national registration number enabled record-linkages between the NLCR and other population-based registers and allowed for a complete, unbiased and long-term follow-up. Taken together, the population-based setting, the completeness, and the high data quality ensured the robustness of our approach. We believe that the use of flexible parametric models incorporating relevant confounders to quantify and illustrate mortality and survival in relative as well as absolute measures is preferential in observational studies of cancer survival.

Several limitations deserve mentioning. Despite the inclusion of a wide range of relevant covariates, we did not have access to individual-level data on every established prognostic factor, and data on *EGFR* and *ALK* mutations was limited to year 2010–2016 and 2016, respectively. All register-based studies may suffer from misclassification, under-reporting and under-diagnosis, it is however unlikely that these biases would differ substantially between men and women. That said, stage migration, due to revision of the TNM classification and introduction of new diagnostic measures in routine work-up, over time is a potential concern [28]. ECOG performance status is based on a subjective assessment of functional activity at diagnosis and possibly affected by gender-related preconceptions. Smoking history was self-reported, vulnerable to response bias, and did not consider number of pack-years. The comorbidity burden was likely underestimated since the Elixhauser groups were based on discharge records from hospitalizations and did not capture comorbidity in patients not admitted to in-hospital care. The inconsistent findings in locally advanced squamous cell carcinoma are difficult to fully explain. It should be noted that this is a heterogeneous group regarding prognosis as well as treatment, and also the smallest subgroup, adding to the difficulty interpreting results.

Our findings corroborate results from prior studies examining sex differences in NSCLC including a higher proportion of adenocarcinoma in women and squamous cell carcinoma in men. As in earlier studies, we found that women with NSCLC were more often never-smokers, diagnosed at an earlier stage and at younger age than men [2, 3, 8, 9, 17, 18]. Our findings of a female survival advantage being particularly pronounced in early stage NSCLC in general and lung adenocarcinoma in particular, have also been reported previously [7, 9, 29–31]. Previous studies further suggested that female sex is an independent favorable prognostic factor in NSCLC [2–7, 11, 13, 16, 32, 33]. The largest study to date included approximately 200,000 lung cancer patients from the SEER database, reported similar findings, but did not include prognostic factors like comorbidity, performance status, smoking history, educational level, and marital status [3]. The few studies reporting comparable or poorer survival in women have been based on small numbers with results likely to have been influenced by substandard treatment in women [34, 35].

In conclusion, we found a higher lung cancer-specific mortality in men with NSCLC that remained largely unchanged following adjustments for a wide range of prognostic factors. Our results provide no evidence of sex differences in the clinical management of NSCLC in Sweden. Taken together, this indicates that the drivers of differences in NSCLC survival between men and women reflect yet to be identified biological sex differences that most probably differ between histological types of NSCLC. With the reservation of limited follow-up time and lacking data on *EGFR* targeted therapy, adjusting for *EGFR* mutational status, a prognostic/predictive factor, in a subgroup analysis did not alter our results. Other known genetic alterations that might differ between men and women, that deserves to be explored further involve DNA repair capacity, *TP53*, *GRPR*, *CYP1A1*, *GSTM1*, *KRAS*, and *ALK*. [17, 36–38]. Estrogen related receptor beta is present in 45–70% pf NSCLC tumors in both sexes, making hormonal influences on tumor biology an appealing hypothesis. [17] The beneficial effect of female sex on prognosis was more or less constant across age group in our data, opposing a female sex hormone influence on survival. While sex differences in treatment toxicity and tumor response provide alternative explanations, earlier studies have not been able to confirm this [4, 32, 39].

Lung cancer is the most commonly diagnosed malignancy as well as cause of cancer death worldwide [40]. Identification and an improved understanding of potentially modifiable factors behind the sex differences in NSCLC survival could potentially prevent or delay a substantial number of cancer deaths. More advanced stage at diagnosis in men could reflect lower health awareness and higher thresholds for seeking health care. Alternatively, observed differences may reflect more aggressive tumor behavior in men, such as faster growth and higher metastatic potential. Irrespective, sex differences in NSCLC biology warrant additional research. In light of the sizable impact of sex on non-small cell lung cancer outcomes, sex should always be reported and results stratified on sex in clinical research.

## Supporting information

S1 Fig Description of subject exclusions. (PDF)

S2 Fig Co-variate effect on the female-to-male hazard ratio. (PDF)

S1 Table Clinical characteristics at diagnosis. *Year of diagnosis 2006–2016. **Year of diagnosis 2003–2016. ***Year of diagnosis 2010–2016.
(PDF)

S2 Table Diagnostic intensity. Numbers (n), percentages (%) of male and female NSCLC patients, and female-to male odds ratios (ORs) with 95% confidence intervals (CI) undergoing diagnostic procedures, by histological type.
^1^Adjusted for age and calendar year of diagnosis. ^2^Additionally adjusted for level of education, marital status, birth country, health care region, ECOG performance status, smoking history, Elixhauser comorbidity categories, TNM stage, and primary tumor location. *Year 2007–2016. **Defined as treatment within 28 days from referral. ***Stage IIIB-IV, year 2010–2016.
(PDF)

S3 Table Lung cancer-specific mortality in pulmonary adenocarcinoma diagnosed in 2010–2016, and tested for *EGFR*. Subgroup analysis of numbers (n), percentages (%) of lung cancer deaths and female-to-male hazard ratios (HRs) with 95% confidence intervals (CI) in men and women diagnosed with pulmonary adenocarcinoma in 2010–2016 and tested for *EGFR* status, by stage group.
HR1: adjusted for age and calendar year of diagnosis. HR2: additionally adjusted for level of education, marital status, birth country, health care region, ECOG performance status, smoking history, Elixhauser comorbidity categories, TNM stage, and primary tumor location. HR3: additionally adjusted for *EGFR* status.
(PDF)

S4 Table Adjusted female-to-male hazard ratios (HR*), exploring interaction. Adjusted female-to-male hazard ratios (HR*) by histological cell type and stage. Exploring the interaction between (i.e. the effect of) female sex and (i.e. on) selected covariates as well as the model fit (p-value) compared to the original model (Table 3).
*Adjusted for age, calendar year, education, marital status, birth country, health care region, ECOG performance status, smoking history, Elixhauser comorbidity groups, TNM stage, and primary tumor location.
(PDF)

S5 Table Comparing 5-year female-to-male hazard ratios estimated using Cox regression and flexible parametric models. Lung cancer specific mortality, 5-year female-to-male hazard ratios by histological cell type and stage, comparing Cox regression (cox) and flexible parametric models (flex).
Model 0: Unadjusted. Model 1: Adjusted for age and calendar year of diagnosis. Model 2: Additionally adjusted for level of education, marital status, birth country, health care region, ECOG performance status, smoking history, Elixhauser comorbidity categories, TNM stage, and primary tumor location.
(PDF)
