# Supplementary material for: Sex and survival in non-small cell lung cancer: A nationwide cohort study
Source: PLoS One. 2019 Jun 27;14(6):e0219206. doi: 10.1371/journal.pone.0219206 (PMC6597110; doi:10.1371/journal.pone.0219206)
Supplement: S1 Fig — (PDF) [file pone.0219206.s001.pdf]

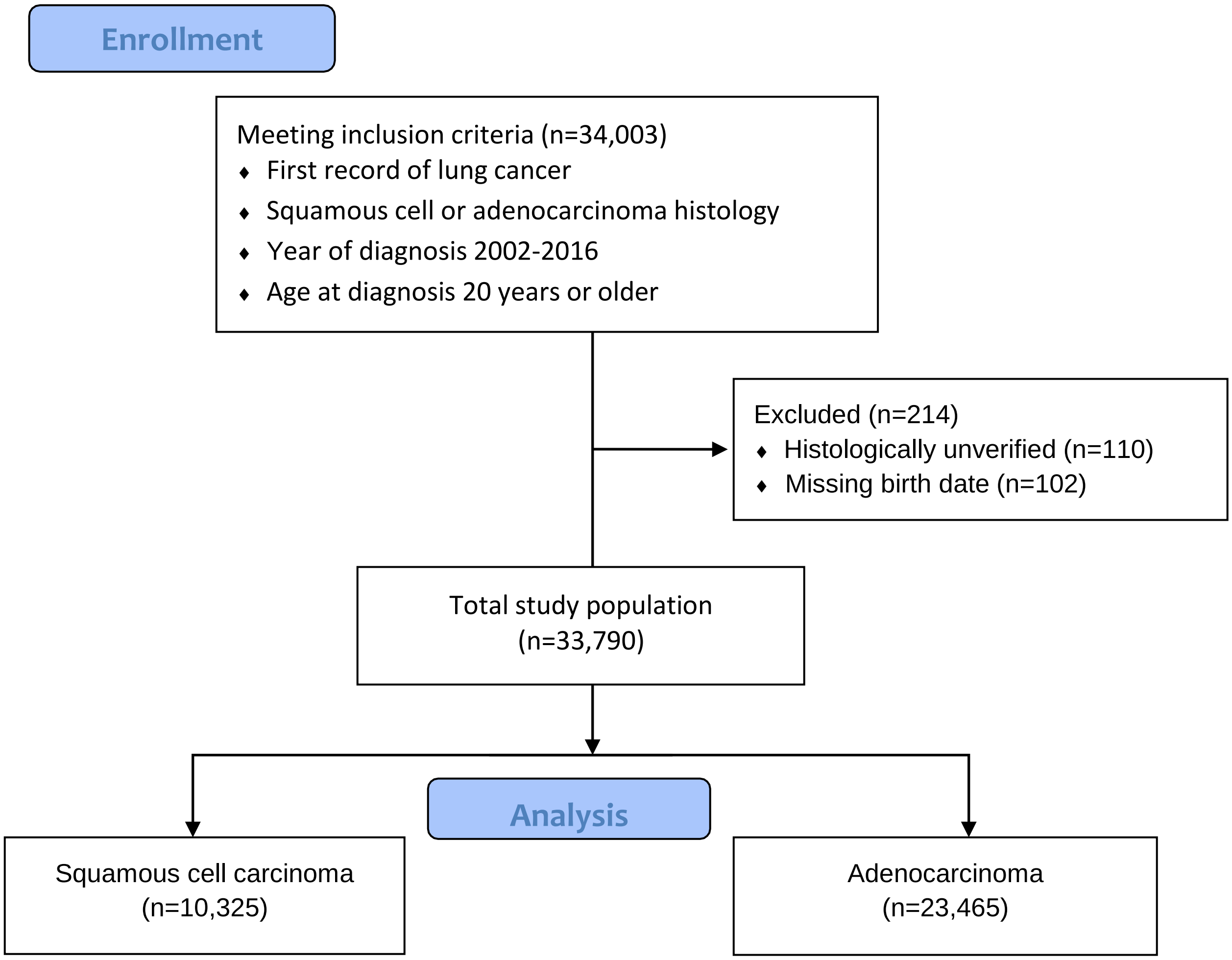

Enrollment
Meeting inclusion criteria (n=34,003)
First record of lung cancer
Squamous cell or adenocarcinoma histology
Year of diagnosis 2002-2016
Age at diagnosis 20 years or older
Excluded (n=214)
Histologically unverified (n=110)
Missing birth date (n=102)
Total study population (n=33,790)
Analysis
Squamous cell carcinoma (n=10,325)
Adenocarcinoma (n=23,465)
