# Supplementary figures and images for: Sex and survival in non-small cell lung cancer: A nationwide cohort study
Source: PLoS One. 2019 Jun 27;14(6):e0219206. doi: 10.1371/journal.pone.0219206 (PMC6597110; doi:10.1371/journal.pone.0219206)

**Supplementary Figure 2A - Squamous cell carcinoma**

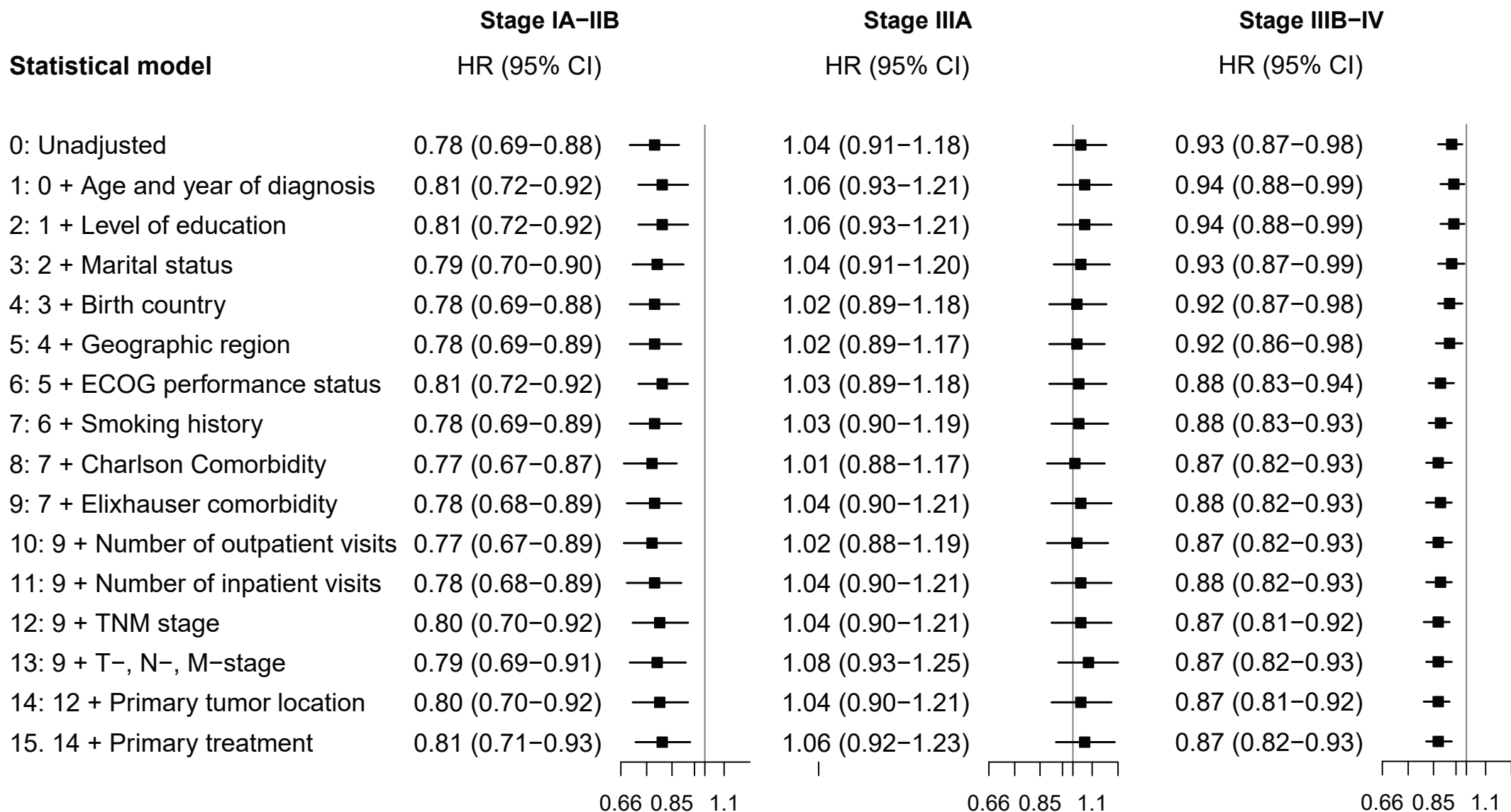

## Supplementary Figure 2B - Adenocarcinoma

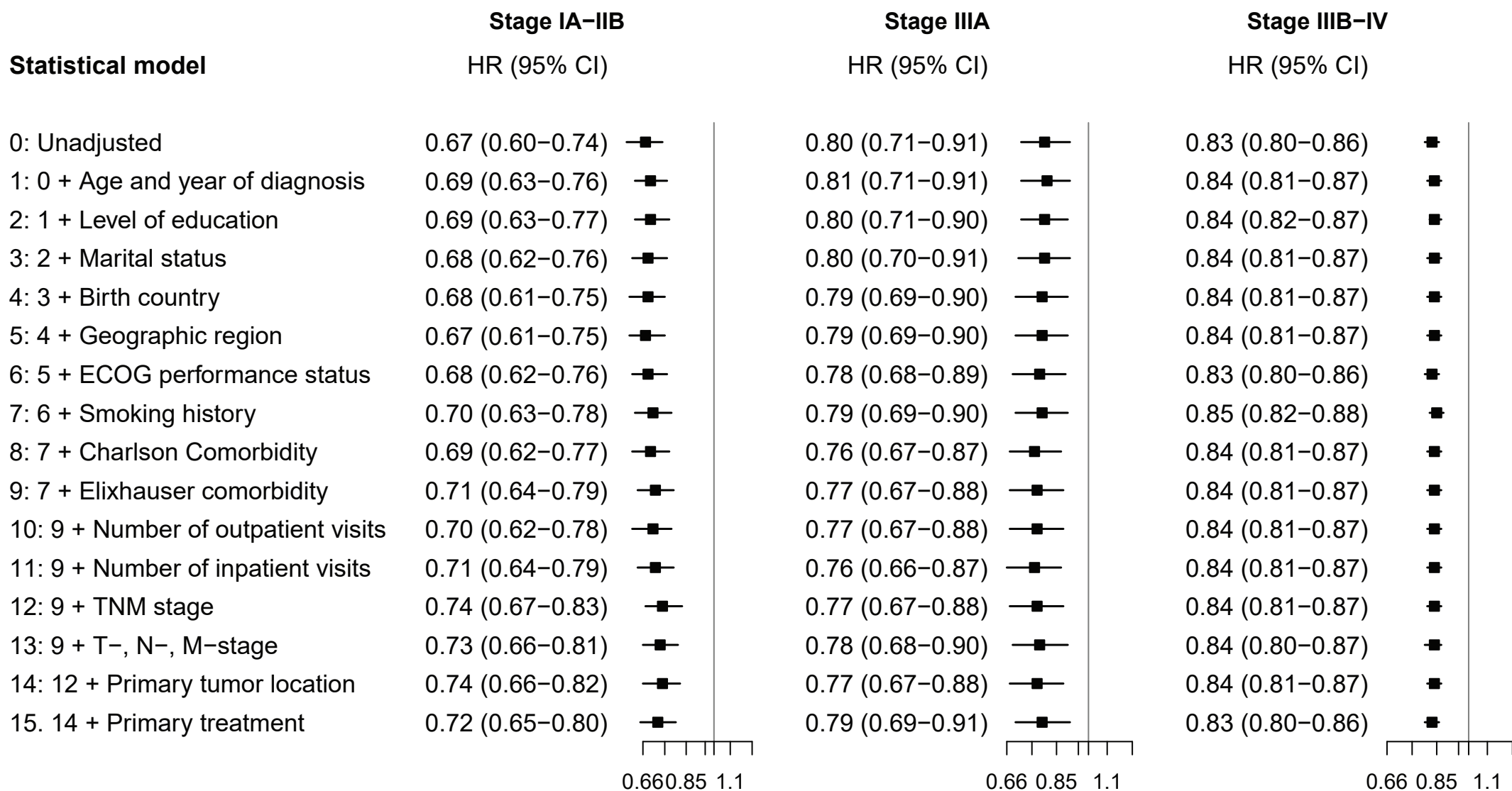

Supplement: S2 Fig — (PDF) [file pone.0219206.s002.pdf]
