# Supplementary material for: Sex and survival in non-small cell lung cancer: A nationwide cohort study
Source: PLoS One. 2019 Jun 27;14(6):e0219206. doi: 10.1371/journal.pone.0219206 (PMC6597110; doi:10.1371/journal.pone.0219206)
Supplement: S1 Table — *Year of diagnosis 2006–2016. **Year of diagnosis 2003–2016. ***Year of diagnosis 2010–2016. (PDF) [file pone.0219206.s003.pdf]

S1 Table. Clinical characteristics at diagnosis.

	Squamous cell carcinoma					Adenocarcinoma				
	Men		Women			Men		Women		
	n	%	n	%	p-value	n	%	n	%	p-value
**All cases**	6556	100.0	3769	100.0		10795	100.0	12670	100.0	
**Calendar period**										
2002-2006	2225	33.9	1119	29.7		2695	25.0	2882	22.7	
2007-2011	2200	33.6	1262	33.5		3671	34.0	4241	33.5	
2012-2016	2131	32.5	1388	36.8	<0.001	4429	41.0	5547	43.8	<0.001
**Education**										
low	3235	49.3	1751	46.5		4559	42.2	4808	37.9	
middle	2383	36.3	1495	39.7		4230	39.2	5427	42.8	
high	762	11.6	452	12.0		1809	16.8	2273	17.9	
missing	176	2.7	71	1.9	<0.001	197	1.8	162	1.3	<0.001
**Marital status**										
married	3663	55.9	1493	39.6		6291	58.3	5497	43.4	
never-married	833	12.7	331	8.8		1366	12.7	1382	10.9	
divorced	1379	21.0	982	26.1		2175	20.1	3070	24.2	
widowed	681	10.4	963	25.6	<0.001	958	8.9	2720	21.5	
missing						5	0.0	1	0.0	<0.001
**Birth country**										

Scandinavia	5808	88.6	3540	93.9		9564	88.6	11830	93.4	
Europe (non-Scandinavia)	581	8.9	185	4.9		827	7.7	602	4.8	
Non-Europe	155	2.4	36	1.0		375	3.5	217	1.7	
missing	12	0.2	8	0.2	<0.001	29	0.3	21	0.2	<0.001
**Health care region**										
Stockholm-Gotland	1076	16.4	646	17.1		2415	22.4	2956	23.3	
Uppsala-Orebro	1472	22.5	828	22.0		2361	21.9	2766	21.8	
South-East	727	11.1	362	9.6		1111	10.3	1284	10.1	
South	1371	20.9	784	20.8		1972	18.3	2259	17.8	
West	1217	18.6	754	20.0		2064	19.1	2356	18.6	
North	693	10.6	395	10.5	0.12	872	8.1	1049	8.3	0.51
**Age**										
0-59	659	10.1	445	11.8		1551	14.4	2344	18.5	
60-69	2002	30.5	1168	31.0		3702	34.3	4571	36.1	
70-79	2711	41.4	1521	40.4		3966	36.7	4106	32.4	
80-89	1148	17.5	612	16.2		1501	13.9	1562	12.3	
90+	36	0.5	23	0.6	0.04	75	0.7	87	0.7	<0.001
**ECOG performance status**										
0	1126	17.2	629	16.7		2501	23.2	3514	27.7	
1	2528	38.6	1553	41.2		3998	37.0	4829	38.1	
2	1502	22.9	875	23.2		2183	20.2	2276	18.0	

3	886	13.5	465	12.3		1256	11.6	1264	10.0	
4	272	4.1	139	3.7		455	4.2	448	3.5	
missing	242	3.7	108	2.9	0.02	402	3.7	339	2.7	<0.001
**Smoking**										
Smoker	3120	47.6	1976	52.4		4109	38.1	5120	40.4	
Former smoker	3160	48.2	1470	39.0		5336	49.4	4957	39.1	
Never smoker	165	2.5	253	6.7		1147	10.6	2384	18.8	
missing	111	1.7	70	1.9	<0.001	203	1.9	209	1.6	<0.001
**CCI**										
0	3115	47.5	1953	51.8		5652	52.4	7614	60.1	
1-2	2280	34.8	1248	33.1		3215	29.8	3336	26.3	
3+	1161	17.7	568	15.1	<0.001	1928	17.9	1720	13.6	<0.001
**Elixhauser comorbidities**										
0	2261	34.5	1386	36.8		4196	38.9	5557	43.9	
1-2	2628	40.1	1469	39.0		4171	38.6	4842	38.2	
3-4	1151	17.6	628	16.7		1703	15.8	1617	12.8	
5+	516	7.9	286	7.6	0.13	725	6.7	654	5.2	<0.001
**Pharmaceutical ATC3 groups***										
0-2	1658	35.0	862	29.9		3292	37.9	3891	37.3	
3-6	2106	44.4	1340	46.4		3817	43.9	4690	45.0	
7+	974	20.6	683	23.7	<0.001	1578	18.2	1848	17.7	0.35

**Outpatient visits prior diagnosis****										
0-1	3868	63.3	2207	61.5		6606	63.6	7767	63.6	
2-4	1429	23.4	863	24.1		2396	23.1	2821	23.1	
5+	818	13.4	518	14.4	0.18	1384	13.3	1632	13.4	1.00
**Inpatient visits prior diagnosis**										
0	5190	79.2	3024	80.2		8816	81.7	10640	84.0	
1	807	12.3	436	11.6		1164	10.8	1295	10.2	
2+	559	8.5	309	8.2	0.42	815	7.5	735	5.8	<0.001
**Stage**										
IA-IIB	1827	27.9	1125	29.8		2470	22.9	3387	26.7	
IIIA	892	13.6	519	13.8		762	7.1	987	7.8	
IIIB-IV	3703	56.5	2069	54.9		7398	68.5	8143	64.3	
missing	134	2.0	56	1.5	0.034	165	1.5	153	1.2	<0.001
**Adrenal metastases**										
yes	286	7.7	178	8.6	0.24	845	11.4	835	10.3	0.02
**Brain metastases**										
yes	274	7.4	209	10.1	<0.001	980	13.2	1295	15.9	<0.001
**Liver metastases**										
yes	427	11.5	248	12.0	0.61	988	13.4	1141	14.0	0.23
**Bone metastases**										
yes	756	20.4	443	21.4	0.37	2186	29.5	2189	26.9	<0.001

**Other metastases**										
yes	1217	32.9	697	33.7	0.52	3078	41.6	3486	42.8	0.13
**Tumor location**										
main bronchus	546	8.3	252	6.7		306	2.8	352	2.8	
upper	3114	47.5	1718	45.6		5461	50.6	6446	50.9	
middle	244	3.7	164	4.4		461	4.3	643	5.1	
lower	2205	33.6	1369	36.3		3518	32.6	4169	32.9	
per continuitatem	97	1.5	54	1.4		165	1.5	162	1.3	
multifocal	184	2.8	114	3.0		594	5.5	610	4.8	
missing	166	2.5	98	2.6	0.01	290	2.7	288	2.3	<0.01
**EGFR mutation*** **										
positive						221	5.5	472	10.3	
negative						1880	46.5	2159	47.1	
inconclusive						157	3.9	210	4.6	
not arrived						23	0.6	34	0.7	
missing/not tested						1765	43.6	1713	37.3	<0.001

*Year of diagnosis 2006-2016. **Year of diagnosis 2003-2016. ***Year of diagnosis 2010-2016.
