# Supplementary material for: Sex and survival in non-small cell lung cancer: A nationwide cohort study
Source: PLoS One. 2019 Jun 27;14(6):e0219206. doi: 10.1371/journal.pone.0219206 (PMC6597110; doi:10.1371/journal.pone.0219206)
Supplement: S2 Table — Numbers (n), percentages (%) of male and female NSCLC patients, and female-to male odds ratios (ORs) with 95% confidence intervals (CI) undergoing diagnostic procedures, by histological type. 1Adjusted for age and calendar year of diagnosis. 2Additionally adjusted for level of education, marital status, birth country, health care region, ECOG performance status, smoking history, Elixhauser comorbidity categories, TNM stage, and primary tumor location. *Year 2007–2016. **Defined as treatment within 28 days from referral. ***Stage IIIB-IV, year 2010–2016. (PDF) [file pone.0219206.s004.pdf]

S2 Table. Diagnostic intensity.

	Squamous cell carcinoma						
	Men			Women			
	n	%	OR (95% CI)	n	%	OR (95% CI)[1]	aOR (95% CI)[2]
Bronchoscopy	5413	82.6	1.00 (ref.)	3011	79.9	0.84 [0.76,0.94]	0.86 [0.76,0.96]
CT thorax	6398	97.6	1.00 (ref.)	3676	97.5	0.97 [0.73,1.28]	0.89 [0.66,1.22]
US/CT abdomen	5813	88.7	1.00 (ref.)	3317	88.0	0.95 [0.84,1.08]	0.97 [0.84,1.11]
Thoracentesis	421	6.4	1.00 (ref.)	205	5.4	0.84 [0.71,1.00]	0.83 [0.68,1.00]
Transthoracic biopsy	1459	22.3	1.00 (ref.)	1075	28.5	1.37 [1.25,1.51]	1.32 [1.19,1.47]
CT/MRI brain*	1021	23.6	1.00 (ref.)	607	22.9	0.93 [0.83,1.05]	0.94 [0.82,1.07]
PET scan*	1978	45.7	1.00 (ref.)	1305	49.2	1.10 [0.99,1.22]	1.07 [0.94,1.22]
Multidisciplinary case conference	4207	64.2	1.00 (ref.)	2506	66.5	1.03 [0.94,1.13]	1.00 [0.90,1.12]
Treatment on time**	2375	36.2	1.00 (ref.)	1331	35.3	0.98 [0.90,1.07]	1.02 [0.92,1.12]
	**Adenocarcinoma**						
	**Men**			**Women**			
	n	%	OR (95% CI)	n	%	OR (95% CI)[1]	aOR (95% CI)[2]
Bronchoscopy	7487	69.4	1.00 (ref.)	8886	70.1	1.03 [0.97,1.09]	1.01 [0.95,1.08]
CT thorax	10531	97.6	1.00 (ref.)	12343	97.4	0.84 [0.71,1.00]	0.82 [0.67,0.99]
US/CT abdomen	9590	88.8	1.00 (ref.)	11341	89.5	1.06 [0.97,1.15]	1.07 [0.98,1.18]
Thoracentesis	1967	18.2	1.00 (ref.)	1903	15.0	0.82 [0.76,0.88]	0.83 [0.76,0.90]
Transthoracic biopsy	3191	29.6	1.00 (ref.)	3722	29.4	0.99 [0.93,1.05]	0.96 [0.90,1.02]
CT/MRI brain*	2041	25.2	1.00 (ref.)	2424	24.8	0.95 [0.89,1.02]	1.01 [0.94,1.10]
PET scan*	3367	41.6	1.00 (ref.)	4460	45.6	1.15 [1.09,1.23]	1.04 [0.95,1.12]
Multidisciplinary case conference	6869	63.6	1.00 (ref.)	8455	66.7	1.08 [1.02,1.14]	1.00 [0.93,1.07]
Treatment on time**	4034	37.4	1.00 (ref.)	4630	36.5	0.94 [0.89,0.99]	0.98 [0.92,1.04]
EGFR testing***	3263	53.4	1.00 (ref.)	4235	57.3	1.11 [1.03,1.19]	1.10 [1.01,1.20]

Numbers (n), percentages (%) of male and female NSCLC patients, and female-to male odds ratios (ORs) with 95% confidence intervals (CI) undergoing diagnostic procedures, by histological type.

[1]Adjusted for age and calendar year of diagnosis. [2]Additionally adjusted for level of education, marital status, birth country, health care region, ECOG performance status, smoking history, Elixhauser comorbidity categories, TNM stage, and primary tumor location. *Year 2007-2016. **Defined as treatment within 28 days from referral. ***Stage IIIB-IV, year 2010-2016.
