# Supplementary material for: Sex and survival in non-small cell lung cancer: A nationwide cohort study
Source: PLoS One. 2019 Jun 27;14(6):e0219206. doi: 10.1371/journal.pone.0219206 (PMC6597110; doi:10.1371/journal.pone.0219206)
Supplement: S3 Table — Subgroup analysis of numbers (n), percentages (%) of lung cancer deaths and female-to-male hazard ratios (HRs) with 95% confidence intervals (CI) in men and women diagnosed with pulmonary adenocarcinoma in 2010–2016 and tested for EGFR status, by stage group. HR1: adjusted for age and calendar year of diagnosis. HR2: additionally adjusted for level of education, marital status, birth country, health care region, ECOG performance status, smoking history, Elixhauser comorbidity categories, TNM stage, and primary tumor location. HR3: additionally adjusted for EGFR status. (PDF) [file pone.0219206.s005.pdf]

S3 Table. Lung cancer-specific mortality in pulmonary adenocarcinoma diagnosed in 2010-2016, and tested for EGFR.

		n	%	HR1 (95% CI)	HR2 (95% CI)	HR3 (95% CI)
**Stage IA-IIB**						
	Men	149	22.6	1.00	1.00	1.00
	Women	157	16.8	0.64 [0.51,0.80]	0.65 [0.50,0.85]	0.65 [0.50,0.85]
**Stage IIIA**						
	Men	138	49.3	1.00	1.00	1.00
	Women	177	46	0.88 [0.70,1.10]	0.91 [0.69,1.18]	0.93 [0.71,1.21]
**Stage IIIB-IV**						
	Men	1665	73	1.00	1.00	1.00
	Women	2012	70	0.85 [0.79,0.90]	0.88 [0.82,0.94]	0.90 [0.84,0.97]

Subgroup analysis of numbers (n), percentages (%) of lung cancer deaths and female-to-male hazard ratios (HRs) with 95% confidence intervals (CI) in men and women diagnosed with pulmonary adenocarcinoma in 2010-2016 and tested for EGFR status, by stage group.

HR1: adjusted for age and calendar year of diagnosis. HR2: additionally adjusted for level of education, marital status, birth country, health care region, ECOG performance status, smoking history, Elixhauser comorbidity categories, TNM stage, and primary tumor location. HR3: additionally adjusted for EGFR status.
