# Supplementary material for: Sex and survival in non-small cell lung cancer: A nationwide cohort study
Source: PLoS One. 2019 Jun 27;14(6):e0219206. doi: 10.1371/journal.pone.0219206 (PMC6597110; doi:10.1371/journal.pone.0219206)
Supplement: S4 Table — Adjusted female-to-male hazard ratios (HR*) by histological cell type and stage. Exploring the interaction between (i.e. the effect of) female sex and (i.e. on) selected covariates as well as the model fit (p-value) compared to the original model (Table 3). *Adjusted for age, calendar year, education, marital status, birth country, health care region, ECOG performance status, smoking history, Elixhauser comorbidity groups, TNM stage, and primary tumor location. (PDF) [file pone.0219206.s006.pdf]

S4 Table. Adjusted female-to-male hazard ratios (HR*), exploring interaction.

	Squamous cell carcinoma						Adenocarcinoma					
	stage IA-IIB		Stage IIIA		Stage IIIB-IV		stage IA-IIB		Stage IIIA		Stage IIIB-IV	
	HR*	P-value	HR*	P-value	HR*	P-value	HR*	P-value	HR*	P-value	HR*	P-value
**Age**												
0-59	0.7515		1.4525		0.9925		0.7089		0.8678		0.9608	
60-69	0.8990		0.9113		0.7823		0.6931		0.8460		0.7799	
70-79	0.7821		0.9321		0.8769		0.7211		0.7317		0.8018	
80-89	0.7311		1.3165		0.8888		0.7639		0.7278		0.9741	
90+	1.9299	0.455	1.8839	0.1503	1.6976	0.0570	1.2583	0.8757	5.974e-06	0.6762	0.6186	0.0000
**Year of diagnosis**												
2002-2006	0.8510		0.9362		0.8176		0.7509		0.6831		0.8481	
2007-2011	0.8087		1.0863		0.9086		0.7165		0.7318		0.8228	
2012-2016	0.7313	0.646	1.0703	0.6483	0.8610	0.3491	0.6869	0.7909	0.9311	0.1141	0.8491	0.7005
**Education**												
low	0.7923		1.2874		0.8715		0.7268		0.8014		0.8742	
middle	0.8708		0.9334		0.8208		0.6844		0.7780		0.8177	
high	0.6722		0.6709		0.9703		0.7813		0.7979		0.8152	
missing	0.7759	0.706	0.6306	0.0096	0.8960	0.4086	0.8036	0.8324	0.7060	0.9938	0.7412	0.2412
**Origin**												
Scandinavia	0.7777		1.0098		0.8596		0.7262		0.8014		0.8425	
Europe	1.2380		1.1466		0.9871		0.7667		0.6294		0.7634	
Non-Europe	3.2384	0.0173	2.4069		0.5565		0.1435		0.7691	0.6895	0.8730	
missing	-		1.7595	0.4564	0.9848	0.3232	0.6073	0.0576	-		1.0161	0.5763
**ECOG performance status**												
0	0.6732		0.8614		0.8039		0.7119		0.8125		0.8560	
1	0.8150		1.0568		0.8797		0.7012		0.8144		0.8423	
2	0.7883		0.9888		0.9357		0.9217		0.7912		0.8521	
3	1.1353		1.1209		0.8906		0.6105		0.4943		0.8279	

4	1.0215		2.5253		0.5093		0.2335		1.6591		0.8599	
missing	0.7199	0.418	1.2728	0.5128	0.9325	0.0008	0.4943	0.1059	0.4156	0.2640	0.6752	0.3034
**Smoking history**												
Smoker	0.7935		1.1757		0.8469		0.7527		0.9623		0.7947	
Former smoker	0.8033		0.8885		0.8714		0.6895		0.6462		0.8422	
Never smoker	0.6222		0.9767		0.8958		0.7239		0.7865		0.9546	
missing	1.8766	0.179	1.1998	0.2634	1.0709	0.7533	0.6592	0.8786	1.1118	0.0310	0.9292	0.0101
**Elixhauser comorbidities**												
0	0.7713		0.9820		0.8745		0.8557		0.8094		0.8353	
1-2	0.8180		1.0126		0.8553		0.6572		0.7673		0.8252	
2-3	0.8456		0.9410		0.8551		0.7486		0.7080		0.8972	
5+	0.7998	0.962	1.7345	0.1665	0.8496	0.9866	0.5520	0.0569	1.0178	0.6583	0.8377	0.5628

Adjusted female-to-male hazard ratios (HR*) by histological cell type and stage. Exploring the interaction between (i.e. the effect of) female sex and selected covariates as well as the model fit (p-value) compared to the original model (Table 3).

*Adjusted for age, calendar year, education, marital status, birth country, health care region, ECOG performance status, smoking history, Elixhauser comorbidity groups, TNM stage, and primary tumor location.
