# Supplementary material for: Sex and survival in non-small cell lung cancer: A nationwide cohort study
Source: PLoS One. 2019 Jun 27;14(6):e0219206. doi: 10.1371/journal.pone.0219206 (PMC6597110; doi:10.1371/journal.pone.0219206)
Supplement: S5 Table — Lung cancer specific mortality, 5-year female-to-male hazard ratios by histological cell type and stage, comparing Cox regression (cox) and flexible parametric models (flex). Model 0: Unadjusted. Model 1: Adjusted for age and calendar year of diagnosis. Model 2: Additionally adjusted for level of education, marital status, birth country, health care region, ECOG performance status, smoking history, Elixhauser comorbidity categories, TNM stage, and primary tumor location. (PDF) [file pone.0219206.s007.pdf]

S5 Table. Comparing 5-year female-to-male hazard ratios estimated using Cox regression and flexible parametric models.

	Squamous cell					
	cox0	flex0	cox1	flex1	cox2	flex2
**Stage IA-IIB**	.7803481	.78012005	.81518691	.81488202	.80230535	.80222086
**Stage IIIA**	1.0360156	1.0360651	1.0605728	1.0610387	1.042404	1.0420136
**Stage IIIB-IV**	.92792317	.92730664	.93735632	.93668126	.86692731	.86605879
	**Adenocarcinoma**					
	cox0	flex0	cox1	flex1	cox2	flex2
**Stage IA-IIB**	.66606878	.66594969	.69109127	.69094193	.73697056	.73649557
**Stage IIIA**	.80774896	.80333501	.81026826	.8063506	.77295858	.76827615
**Stage IIIB-IV**	.83189153	.83144136	.84339507	.84299266	.83708629	.83633157

Lung cancer specific mortality, 5-year female-to-male hazard ratios by histological cell type and stage, comparing Cox regression (cox) and flexible parametric models (flex).

Model 0: Unadjusted. Model 1: Adjusted for age and calendar year of diagnosis. Model 2: Additionally adjusted for level of education, marital status, birth country, health care region, ECOG performance status, smoking history, Elixhauser comorbidity categories, TNM stage, and primary tumor location.
